# Feasibility and correlation of standard 2D speckle tracking echocardiography and automated function imaging derived parameters of left ventricular function during dobutamine stress test

**DOI:** 10.1007/s10554-014-0386-z

**Published:** 2014-02-13

**Authors:** Karina Wierzbowska-Drabik, Piotr Hamala, Nikolina Roszczyk, Piotr Lipiec, Michał Plewka, Radosław Kręcki, Jarosław Damian Kasprzak

**Affiliations:** Chair and Department of Cardiology, Medical University of Lodz, Kniaziewicza 1/5, 91-347 Lodz, Poland

**Keywords:** Dobutamine stress echocardiography, Speckle tracking, Automated function imaging, Systolic longitudinal strain

## Abstract

Speckle tracking echocardiography (STE) is a method of quantitative assessment of myocardial function complementary to ejection fraction and visual evaluation. Standard STE analysis, demands manual tracing of the myocardium whereas automated function imaging (AFI) offers more convenient (based on selection of three points) assessment of longitudinal strain. Nevertheless, feasibility and correlation between both methods were not thoroughly examined, especially during tachycardia at peak stage of dobutamine stress echocardiography (DSE). We performed DSE in 238 patients (pts) with recording of apical views during baseline (0) and peak (1) DSE and analyzed them by STE and AFI. According to angiography, 127/238 pts had significant (≥70 %) lesions in coronary arteries. We assessed correlations between STE and AFI derived peak systolic longitudinal strain values for global and regional parameters, feasibility, time of analysis and interobserver agreement. Global systolic longitudinal strain measured during baseline and peak stage of DSE by AFI showed very good correlation with standard STE parameters, with correlation coefficients r = 0.90 and r = 0.86 respectively (*p* < 0.0001). For regional parameters correlation coefficients ranged from 0.83 to 0.85 for baseline and from 0.70 to 0.79 for peak DSE. Both methods provided good and similar feasibility with only 1 % segments excluded from analysis at peak stage of DSE with shorter time and lower coefficient of variance offered by AFI. Global and regional longitudinal strain achieved by faster and less operator-dependent AFI method correlate well with standard more time-consuming STE analysis during baseline and peak stage of DSE.

## Introduction

Stress echocardiography is, besides nuclear imaging, one of the leading noninvasive tests widely applied in the diagnosis of coronary artery disease [1, 2]. The proven diagnostic advantage over ECG-based detection of ischemia, observed especially in women, is however, limited by subjectivity of visual assessment of regional contractile function. Thus, various methods potentially increasing interobserver agreement and providing quantitative parameters of segmental systolic function are intensely tested [3–8]. The clinical introduction of noninvasive tools for the assessment of myocardial deformation provided the impulse to testing of their utility in the field of stress echocardiography [9]. Since early 90, deformation imaging based on Doppler method has been tested, showing the relationship between ischemia and longitudinal strain rate decrease and discovering the phenomenon of post systolic shortening with possibility of its quantification [10–13]. The application of two-dimensional image (2D)-based speckle tracking echocardiography (STE) allowed to overcome some of drawbacks of Doppler-derived method such as angle-dependency, the general limitation to longitudinal direction of deformation and the lack of possibility of accurate evaluation of the left ventricular apex at the cost of lower time resolution of STE [14]. In spite of wider application and more user-friendly analysis of myocardial deformation with standard STE method, quantitative assessment of regional left ventricular function is still time-consuming and the presentation of results lacks synthetic display. These shortcomings are especially important for the assessment of myocardial function during dobutamine stress echocardiography (DSE) when meticulous evaluation of segmental function should be repeated at least at baseline and peak stage. Thus a new based on STE technique, automated function imaging (AFI) allowing a faster assessment of regional longitudinal strain of the left ventricle and clear results presentation as a polar map has recently gained wide interest and has been tested in various clinical settings although its applicability for DSE is still poorly documented. The aim of our study was to evaluate the relationships of regional and global parameters of left ventricular longitudinal strain derived by standard STE and a novel, semiautomatic AFI method, during baseline and peak stage of DSE and compare feasibility of both methods.

## Methods

### Study group and protocol

We examined 238 subjects (105 women, mean age 62 ± 9, mean left ventricle ejection fraction 59 ± 10 %) with symptoms suggesting stable angina and scheduled for myocardial ischemia testing. The study protocol encompassed: medical history, physical examination, resting ECG and laboratory data (lipoproteins, glucose, and creatinine), transthoracic echocardiographic examination, dobutamine stress echocardiography with early atropine administration (DSE) and angiographic examination in all patients performed no later than 3 months after DSE. The assessment of coronary arteries was done by invasive coronary angiography in 208 patients and by computed tomography in 35 subjects (5 patients had both examinations). As significant lesion stenosis ≥50 % for left main coronary artery (LMCA) and ≥70 % for others epicardial arteries was defined. All study patients were in sinus rhythm and had no significant valve disease (except for mild to moderate insufficiency). Exclusion criteria included contraindications to dobutamine and atropine: acute coronary syndromes, valve or outflow tract stenosis, blood pressure >200/100 mmHg, ventricular tachycardia, glaucoma or prostatic adenoma. Demographic characteristics and echocardiographic data of the examined subjects are displayed in Tables 1 and 2. All subjects gave written informed consent to participate in the study and the protocol was approved by Ethical Commission of Medical University of Lodz (number of agreement RNN/119/10 KE from 13.07.2010).

**Table 1:** Demography, risk factors and treatment of the studied group

Parameter in studied group n = 238	Mean ± SD	Range
Age (years)	62 ± 9	38–61
Height (cm)	168 ± 9	146–187
Body mass (kg)	81 ± 15	43–125
Waist to hip ratio	0.92 ± 0.1	0.64–1.2
Body mass index (kg/m^2^)	28.7 ± 4.7	17.6–46.9
Body surface area (m^2^)	1.94 ± 0.2	1.35–2.52
Hypertension (number %)	207	86
Diabetes mellitus (number %)	66	28
Smoking (number %)	135	56
Hypercholesterolemia (number %)	197	82
Hypertriglyceridemia (number %)	152	63
Family history of CAD (number %)	41	17
History of myocardial infarction (number %)	70	29
Acetylsalicylic acid (number %)	208	87
Clopidogrel (number %)	75	32
Betaadrenolytic (number %)	177	74
ACE inhibitor (number %)	183	76
Statin (number %)	204	86
Long-acting nitrates (number %)	116	49

*CAD* coronary artery disease, *ACE* angiotensin-converting enzyme, *LDL* low density lipoprotein, *HDL* high density lipoprotein

**Table 2:** Characteristic of basic echocardiographic parameters of studied group at baseline

Parameter in studied group n = 238	Mean ± SD	Range
LVd (mm)	47 ± 5	35–61
LVs (mm)	33 ± 5	22–50
PWd (mm)	11 ± 1	8–16
PWs (mm)	14 ± 2	10–19
IVSd (mm)	12 ± 2	8–17
IVSs (mm)	15 ± 2	11–20
Ao (mm)	33 ± 4	25–46
LA (mm)	40 ± 4	30–53
RV (mm)	26 ± 2	20–32
E/A	0.89 ± 0.37	0.4–4.0
LV mass (g)	240 ± 66	102–464
LV mass index (g/m^2^)	123 ± 29	51–254

*LVd* left ventricular diastolic dimension, *LVs* left ventricular systolic dimension, *PWd* diastolic thickness of the left ventricular posterior wall, *PWs* systolic thickness of the left ventricular posterior wall, *IVSd* diastolic thickness of left ventricular septum, *IVSs* systolic thickness of left ventricular septum, *Ao* aortic dimension, *LA* left atrial dimension, *RV* right ventricular dimension, *E/A* ratio of early to atrial mitral inflow peak velocity, *LV mass* left ventricular mass, *LV mass* *index* left ventricular mass index

* *p* < 0.05

**Table 3:** Comparison of heart rate, blood pressure and chosen echocardiographic parameters of studied group between baseline and peak stage of DSE

Parameter in studied group n = 238	Mean ± SD at baseline	Mean ± SD at peak	*p* value
Heart rate DSE (bpm)	66 ± 10	139 ± 17	<0.0001
Blood pressure systolic (mmHg)	129 ± 18	142 ± 25	<0.0001
Blood pressure diastolic (mmHg)	71 + 10	77 ± 16	<0.0001
EF (%)	58 ± 10	66 ± 9	<0.0001
S′ lat (cm/s)	8 ± 2	14 ± 5	<0.0001
E′ lat (cm/s)	10 ± 3	15 ± 4	<0.0001
Wall motion index	1.08 ± 0.24	1.17 ± 0.27	<0.0001
Global SLS by STE (%)	−16.4 ± 3.6	−15.0 ± 4.1	<0.0001
Global SLS by AFI (%)	−17.4 ± 3.9	−16.6 ± 4.3	=0.0003
Average SLS 2 ch by STE (%)	−16.3 ± 4.2	−14.9 ± 4.8	<0.0001
Average SLS 3 ch by STE (%)	−16.3 ± 4.1	−14.7 ± 4.9	<0.0001
Average SLS 4 ch by STE (%)	−16.6 ± 4.0	−15.4 ± 5.2	=0.0001
Average SLS 2 ch by AFI (%)	−17.6 ± 4.4	−16.2 ± 5.0	<0.0001
Average SLS 3 ch by AFI (%)	−17.5 ± 4.6	−16.9 ± 5.4	=0.0536
Average SLS 4 ch by AFI (%)	−17.1 ± 4.3	−16.5 ± 5.1	=0.0328

*DSE* dobutamine stress echocardiography, *LVEDV* end diastolic volume of left ventricle, *LVESV* end systolic volume of left ventricle, *EF* ejection fraction, *S′lat* peak velocity of lateral part of mitral annulus in systolic phase, *E′lat* peak velocity of lateral part of mitral annulus in diastolic phase, *SLS* systolic longitudinal strain, *STE* speckle tracking echocardiography, *AFI* automated function imaging, *2 ch* apical two-chamber view, *3 ch* apical three-chamber view, *4 ch* apical four-chamber view

### Echocardiography at rest and during DASE

Transthoracic echocardiography was performed with VIVID 7 Dimension (GE Vingmed Ultrasound AS, Horten, Norway) using M4S probe in harmonic mode 2.0/4.3 MHz with maximal frame per second (FPS) count available at necessary sector width. Range of FPS was from 64 to 112 with mean value 83. The assessment of left ventricular (LV) systolic and diastolic volumes and ejection fraction was done using a modified Simpson’s method from triplane view obtained with volumetric probe 3 V. Mass of LV was calculated from Devereaux’s formula with measurements of LV walls and cavity was performed according to Penn convention. Wall thickness and chamber dimensions were measured from 2D imaging in parasternal long axis view. The echocardiographic measurements were done following American Society of Echocardiography/European Association of Echocardiography guidelines. The assessment of left ventricular contractility was done visually by two experts and classified for each segment as norm kinesis, hypokinesis, akinesis or diakinesis. 18-segments model of left ventricle was applied dividing each of LV wall visualized from apical projections into three segments: basal, mid and apical. The worsening of contractility in at least two adjacent segments of left ventricle was consistent with a positive dobutamine stress test. Early atropine administration protocol was applied. Dobutamine was administered in the intravenous infusion in doses of 10, 20, 30 and 40 μg/kg/min during 3-min stages whereas atropine was added in 0.5 mg fractional doses after the second stage of infusion up to the total dose of 2 mg. The infusion of dobutamine was stopped when age-adjusted HR limit, positive stress test result or other criteria for terminating DSE were fulfilled. For safety reasons, the presence of minimum two persons during each study was mandatory, BP was measured during each stage of test, 12-lead ECG was recorded immediately after stopping dobutamine infusion and a prolonged observation up to 1 h after the test was scheduled.

### The assessment of deformation

**Fig. 1:**
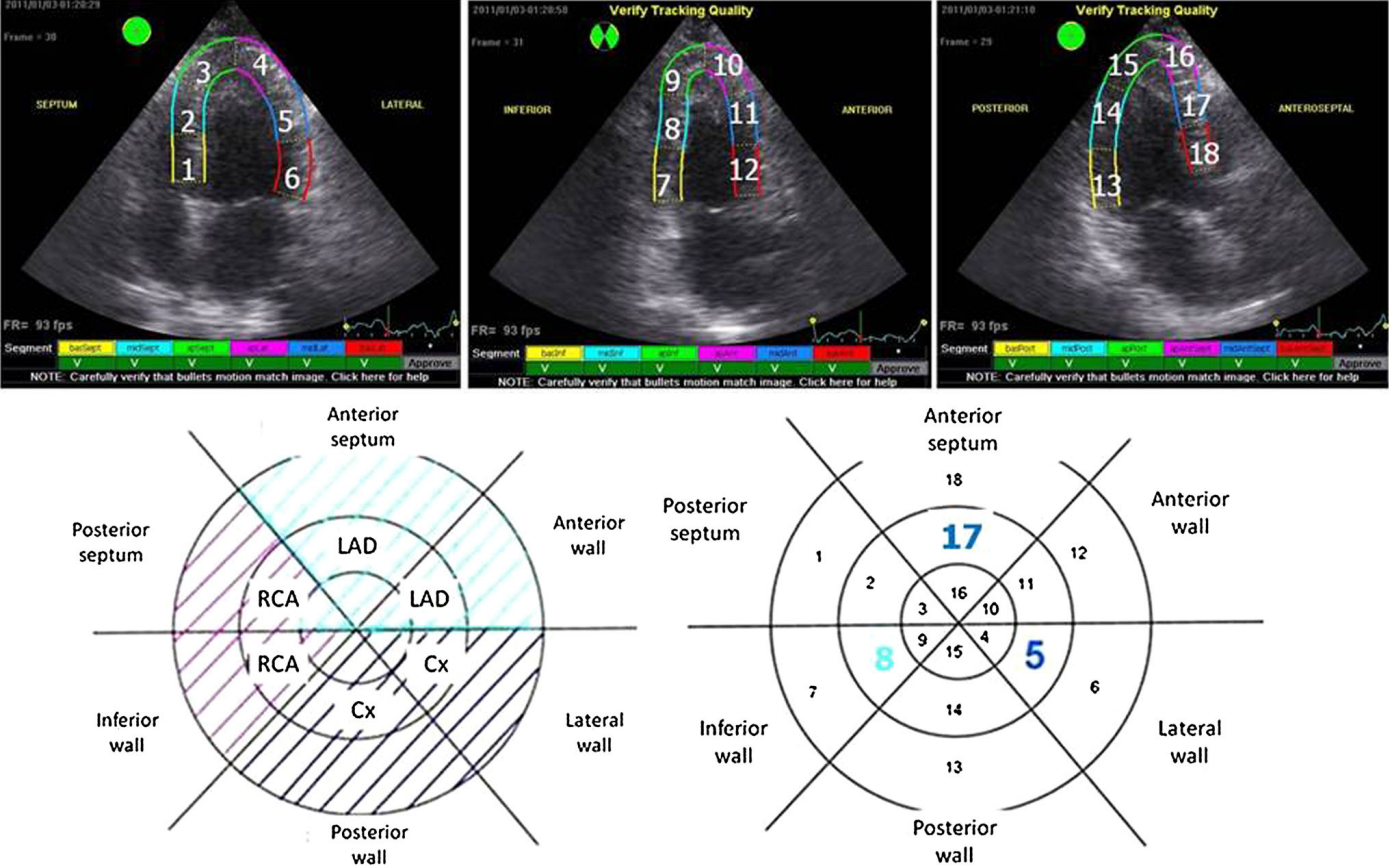
The annotation of the left ventricular segments and definition of regions supplied by respective coronary arteries

Digital loops from standard echocardiographic views (three apical views and LV short axis views at three levels- mitral valve, papillary muscle and apical) were digitally stored at baseline and peak stage of the infusion for further analysis. The calculation of regional and global parameters of deformation was done off-line using EchoPac 6.1.0 workstation (GE Vingmed Ultrasound). The allocation of regional values of longitudinal strain by standard 2D speckle tracking imaging required manual marking of endocardial contour and adjustment of the region of interest to encompass the myocardial wall in all three apical views. For the analysis, we assigned left ventricular segments with consecutive numbers in clockwise order starting from basal septum in 4-chamber view, through 2-chamber to 3-chamber view. Mid-segments from lateral wall, inferior wall and anterior septum were chosen as marker segments for the circumflex, right coronary artery and left anterior descending artery territories respectively. The annotation of the myocardial segments and definition of region supplied by respective coronary arteries are displayed in Fig. 1. For each LV segment the value of peak systolic longitudinal strain was measured at baseline and peak level of dobutamine stress test (SLS_0_ and SLS_1_ respectively, systolic longitudinal strain). Additionally, the averaged values of SLS for each apical view were measured and global SLS from 18 left ventricle segments was calculated. The obtaining of regional and global systolic longitudinal strain by AFI method demanded manual marking of three points (two on basal and one on apical endocardium) in each apical view and accepting the resulting region of interest. The rounded segmental values of peak systolic longitudinal strain were displayed in a straightforward format of polar map with additional information regarding the averaged (obtained from six segments) and global (obtained from 18 segments) systolic longitudinal strain.

The parameters measured by both techniques were defined as the peak deformation in longitudinal direction achieved for assessed segment during systole, before the aortic valve closure. Correlations were calculated for global and regional longitudinal strain in all segments.

### Statistical analysis

Statistical analysis was performed using MedCalc V. 12.1.4. (Frank Schoonjans Belgium). Continuous variables were expressed as means and standard deviations. Mean values were compared with *t* Student test for paired variables. Chi square test was used to test the dichotomous variables distribution. For comparison of parameters obtained by STE and AFI Pearson correlation coefficients were calculated and Bland–Altman analysis performed. For interobserver variability assessment we calculated coefficients of variance. Duration of STE and AFI calculations and interobserver agreement were assessed in the group of 12 randomly selected subjects.

## Results

Time needed to analyze and obtain regional, averaged (from six segments) and global (from 18 segments) systolic longitudinal strain by classic STE was 367 ± 39 s and by AFI 168 ± 28 s, the difference was statistically significant with *p* < 0.0001. Interobserver variability (coefficient of variance) calculated for regional longitudinal strain in segment number one (basal septum) was 8.7 and 16 % respectively for AFI at baseline and peak stage of DSE and for STE 13.3 and 24.2 %.

To our study we included only subjects (n = 238) in which visual assessment of endocardium was feasible in all left ventricular segments (n = 4,284 segments), we excluded—six patients (2.5 %) from the study because of insufficient visualization. Suboptimal quality of 2D image—as assessed automatically by software despite manual correction of region of interest- led to exclusion of 11 segments (0.26 %) from STE and 18 segments (0.42 %) from AFI analysis at rest. During the peak stage of DSE 41 segments (0.96 %) were suboptimal for STE analysis and 46 (1.07 %) were suboptimal for AFI quantification and excluded from the analysis. For both methods proportion of segments with suboptimal quality was significantly higher at peak stage of stress test than at baseline (*p* = 0.0001 for STE and *p* = 0.0007 for AFI) whereas the differences of feasibility between STE and AFI respectively at baseline and stress were not significant.

**Fig. 2:**
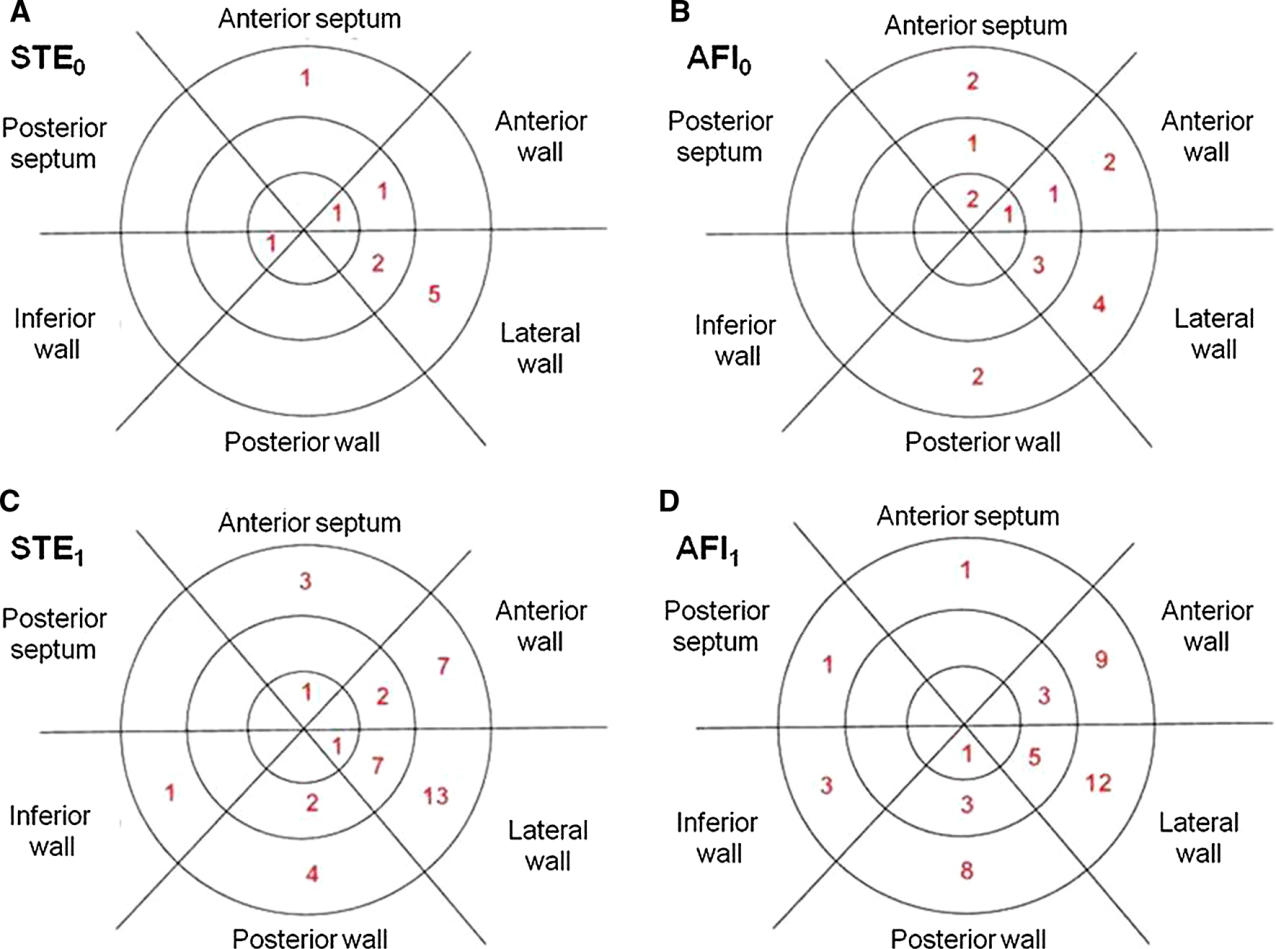
The number of segments excluded from analysis by STE and AFI. Polar maps display regional dispersion of feasibility—with sum of 45 excluded segments in both stages of DSE in the region of anterior, lateral and posterior wall in analysis using STE versus 7 segments in region of septum and inferior wall. From AFI this proportion was similar with 54 versus 10 excluded segments in respective regions of left ventricle. *STE*_*0*_ number of segments excluded from strain measurements by STE at rest, *AFI*_*0*_ number of segments excluded from strain measurements by AFI at rest, *STE*_*1*_ number of segments excluded from strain measurements by STE at peak stress test, *AFI*_*1*_ number of segments excluded from strain measurements by AFI at peak stress test

The feasibility of longitudinal systolic strain analysis at rest and stress calculated for individual left ventricular segments (range from 94.5 to 100 %) is displayed in Table 4. Figure 2 presents numbers and localization of excluded segments in polar maps of the left ventricle.

**Table 4:** The feasibility of longitudinal strain analysis estimation by STE and AFI in all left ventricular segments at rest and peak stage of DSE

Segment	STE_0_ (%)	AFI_0_ (%)	STE_1_ (%)	AFI_1_ (%)
Basal posterior septum (seg. 1)	100	100	100	99.6
Mid posterior septum (seg. 2)	100	100	100	100
Apical posterior septum (seg. 3)	100	100	100	100
Apical lateral wall (seg. 4)	100	100	99.6	100
Mid lateral wall (seg. 5)	99.2	98.7	97.1	97.9
Basal lateral wall (seg. 6)	97.9	98.3	94.5	95
Basal inferior wall (seg. 7)	100	100	99.6	98.7
Mid inferior wall (seg. 8)	100	100	100	100
Apical inferior wall (seg. 9)	99.6	100	100	100
Apical anterior wall (seg. 10)	99.6	99.6	100	100
Mid anterior wall (seg. 11)	99.6	99.6	99.2	98.7
Basal anterior wall (seg. 12)	100	99.2	97.1	96.2
Basal posterior wall (seg. 13)	100	99.2	98.3	96.6
Mid posterior wall (seg. 14)	100	100	99.2	98.7
Apical posterior wall (seg. 15)	100	100	100	99.6
Apical anterior septum (seg. 16)	100	99.2	99.6	100
Mid anterior septum (seg. 17)	100	99.6	100	100
Basal anterior septum (seg. 18)	99.6	99.2	98.7	99.6

*STE*_*0*_ feasibility of strain measurements done by STE at rest, *AFI*_*0*_ feasibility of strain measurements done by AFI at rest, *STE*_*1*_ feasibility of strain measurements done by STE at peak stress test, *AFI*_*1*_ feasibility of strain measurements done by AFI at peak stress test

**Fig. 3:**
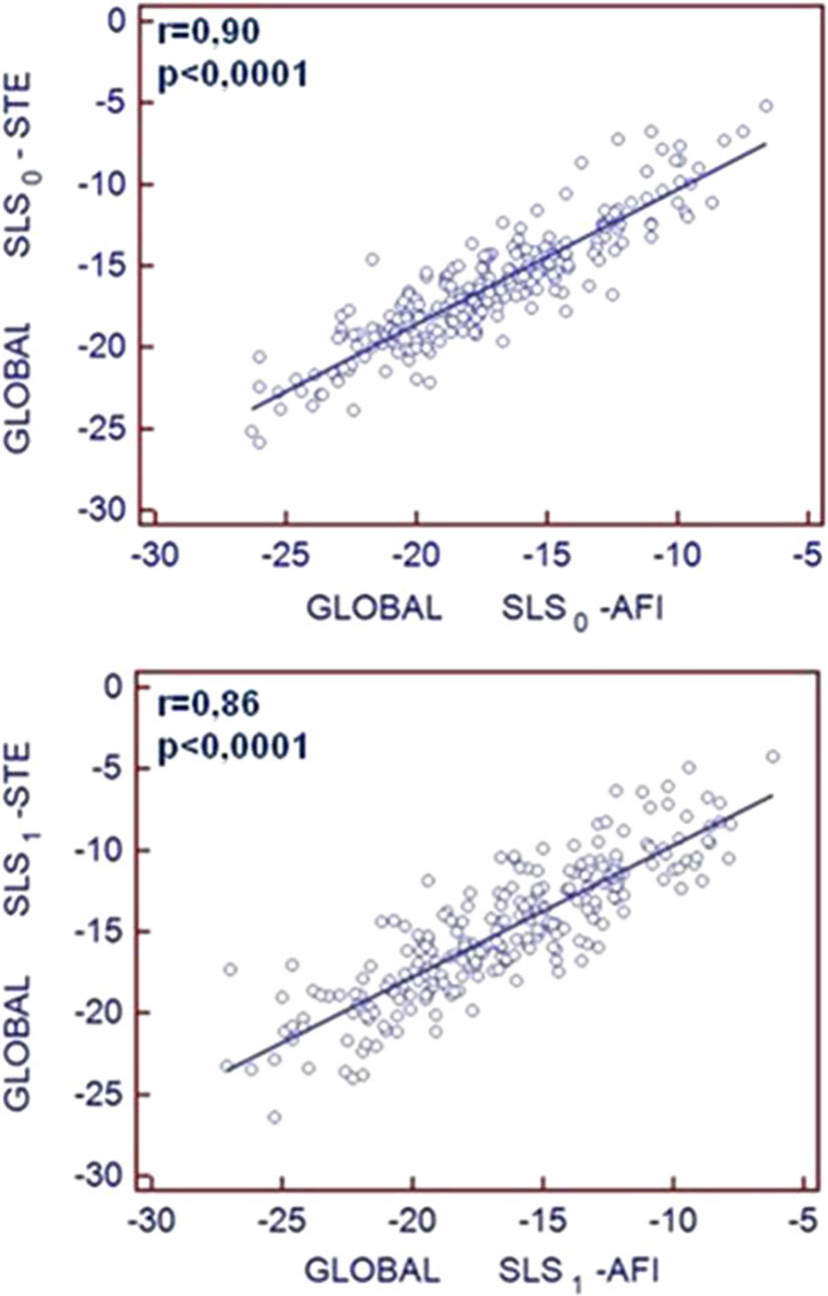
The graphs presenting the significant correlation between systolic longitudinal strain measured by classic speckle tracking echocardiography (STE) and automated function imaging (AFI) during baseline (*upper panel*, index 0) and at peak stage of DSE (*lower panel*, index 1)

**Fig. 4:**
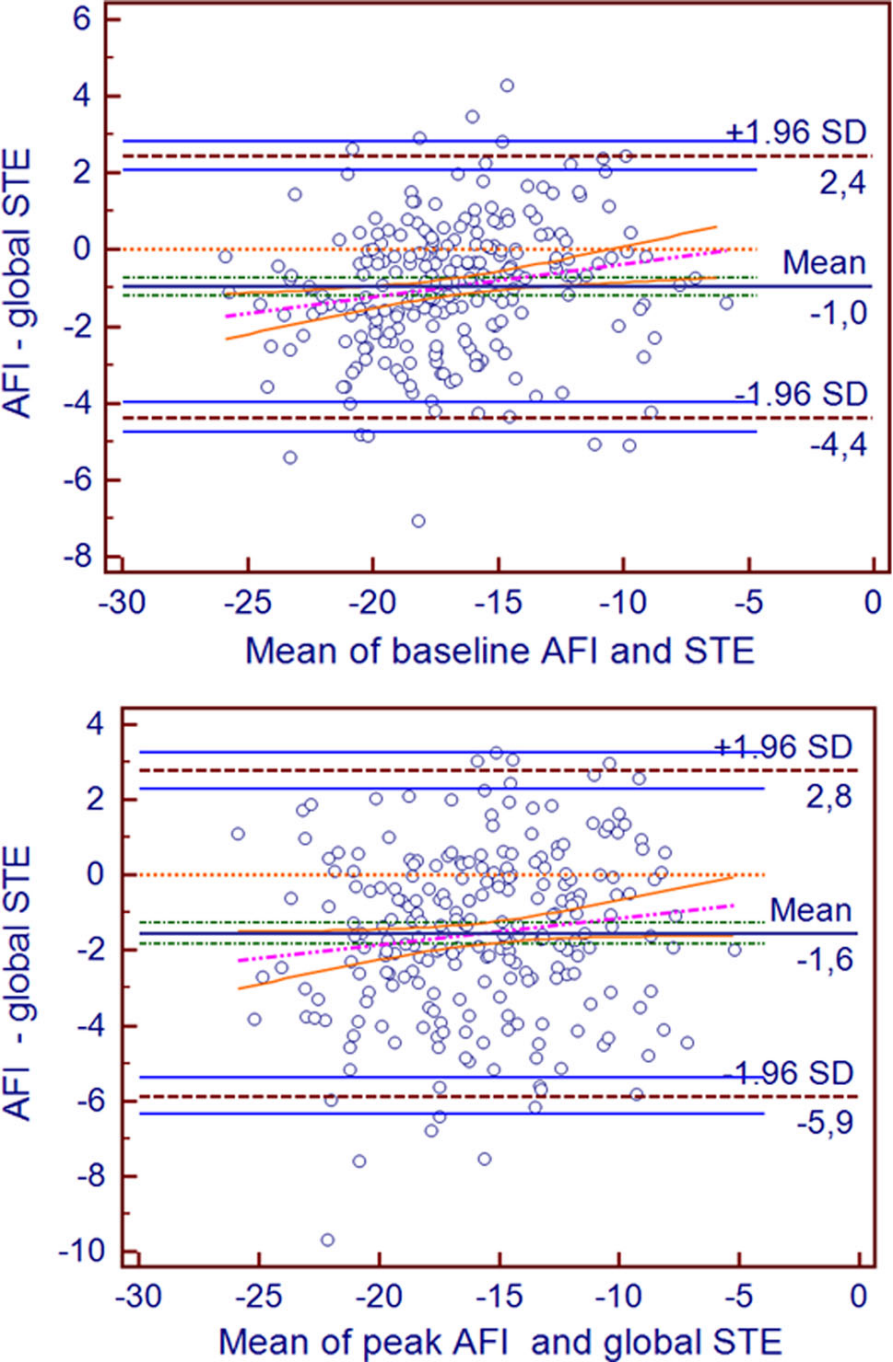
The Bland–Altman analysis of global systolic longitudinal strain measurements achieved by both methods at baseline (*upper panel*) and peak stage. *Dotted orange line*—line of equality, *pink line*—regression line, *orange continuous lines—*confidence intervals of regression line, *green lines—*confidence intervals of mean of difference line, *blue lines—*confidence intervals of limit of agreement lines

Expectedly, dobutamine induced the increase of heart rate, blood pressure, ejection fraction and peak velocities of mitral annulus as summarized in Table 3. The diastolic and systolic volumes of left ventricle and absolute values of longitudinal strain decreased during dobutamine stress test, see Table 3. Global systolic longitudinal strain measured during baseline and peak stage of DSE by AFI showed strong correlation with standard STE parameters (correlation coefficients r = 0.90 and r = v86 respectively, *p* < 0.0001), see Fig. 3. The good agreement of the examined methods for determination of global strain was also confirmed in Bland–Altman analysis, see Fig. 4.

**Fig. 5:**
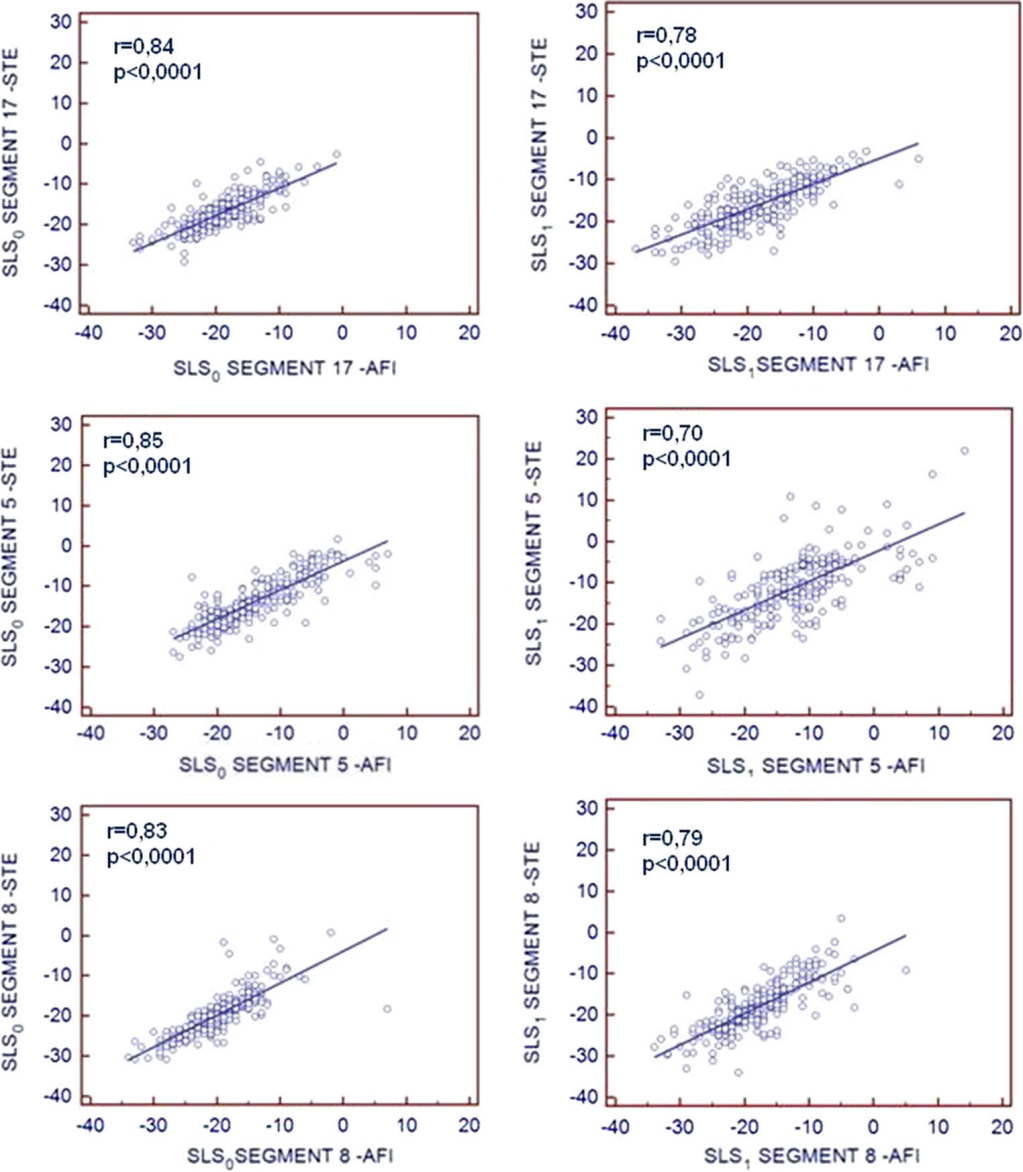
Correlations of peak regional longitudinal strain values measured by STE and AFI during baseline and peak DSE. *SLS*_*0*_ systolic longitudinal strain at baseline, *SLS*_*1*_ systolic longitudinal strain at peak stage of dobutamine test. *STE* speckle tracking echocardiography, *AFI* automated function imaging. Segment 17- mid segment of anterior septum, 5-mid segment of lateral wall, 8- mid segment of inferior wall

The comparison of regional deformation parameters measured in subsequent left ventricular segments indicated highly significant linear relationship of values from both methods (*p* < 0.0001), although correlation coefficients for measurements performed at baseline were slightly higher than during peak stage of dobutamine test. In the Fig. 5 the relationships between systolic longitudinal strain measured by STE and AFI method in three mid-ventricular “marker” segments supplied by left anterior descending, circumflex and right coronary artery, respectively are shown.

## Discussion

Although the application of two-dimensional speckle-tracking in the clinical studies is reaching the decade, and the newest techniques concerns three-dimensional and layer-specific assessment of myocardial function there is still a paucity of data comparing standard 2D STE and its newer modification AFI in the same subset of patients, especially in the setting of stress test [15–17]. Additional concerns were evoked by JUSTICE study indicating lower than expected reproducibility of deformation measurements when compared among different vendors of echocardiographic machines [18]. On the other hand, recently published studies documented the advantage of quantitative assessment of deformation over visual evaluation of regional wall motion of left ventricle and good reproducibility of strain/strain rate parameters at consecutive levels of low-dose dobutamine protocol [19, 20].

In our study we documented the moderate clinical utility of standard 2D STE for the evaluation of left ventricular function during DSE with high percentage of segments amenable to quantification (>90 %) but with limited interobserver agreement, especially when systolic longitudinal strain was measured during significant tachycardia (mean value of heart rate 139 ± 17 beat per minute) at peak stage of test. The high observed feasibility of STE and AFI is comparable to values reported in the literature although the findings in our study may be influenced by preselection of the subjects with all segments suitable for visual assessment [21, 22].

The novel, simplified AFI technique offered similar feasibility, lower coefficient of variance both during rest and peak stress stage (8.7 vs 13.3 % and 16 vs 24.2 % respectively) and significantly (about two times) shorter time needed for analysis. The preponderance of AFI over velocity vector imaging (VVI) -based strain calculations was observed in earlier studies concerning patients with suspected or documented coronary artery disease and using magnetic resonance strain imaging as a golden standard [23].

As expected the localization of segments excluded from analysis was similar for both traditional STE and AFI with lower feasibility in regions of anterior, lateral and posterior wall (see polar maps in Fig. 2) [24].

Despite worse feasibility and repeatability during peak stage of DSE, a highly significant strong correlation between systolic longitudinal strains measured by both methods was preserved not only at rest but also during peak stage of the test (see Figs. 3, 5). Good agreement between both techniques for global left ventricular strain evaluation was also corroborated by Bland–Altman analysis (Fig. 4).

The present clinical standard for contractility evaluation during echocardiographic stress test is visual assessment of wall motion (endocardial displacement and myocardial thickening) performed optimally as expert consensus by experienced and accordingly trained observers and supported by second harmonic imaging, echocardiographic contrast enhancement as needed, digital storage and quad screen format [6, 25, 26]. The visual assessment is yet limited by high interobserver variability and relies mainly on myocardial radial performance, whereas subendocardial longitudinal myocardial layer is the most sensitive to ischemia.

Considering the known limitation of visual assessment of wall motion during echocardiographic stress test, especially those connected with significant tachycardia (such as dobutamine, exercise and rapid pacing) and meticulous analysis necessary in standard STE, the novel AFI method appears faster, offering user-friendly polar maps of the left ventricle and thus a potential to become a useful clinical tool.

## Limitations

The main limitation of our study is a lack of a golden standard for echocardiographic measures of deformation. Magnetic resonance imaging myocardial tagging could be proposed as a reference but its practical use is very limited. Instead, we focused on a direct comparison of two easily amenable echocardiographic modalities.

Another limitation of our study is the exclusion of subjects with the suboptimal echocardiographic visualization assessment of endocardial border. Nevertheless the percentage of subjects excluded since suboptimal quality of visualization was really low (below 3 %) and the number of included examination still exceeded 200 DSE tests with sum >4,000 analyzed segments.

Single-center character and the assessment of STE and AFI correlation confined to a single vendor of echocardiographic equipment may be considered as subsequent limitation of our study as evidence is growing regarding a poor agreement between different STE softwares.

## Conclusions

The quantitative assessment of longitudinal regional and global strain during DSE is feasible by both standard 2D STE and AFI methods. Parameters achieved by those techniques showed strong correlations at rest ant at the peak stage of dobutamine stress test. The shorter time necessary for analysis by AFI method and lower operator-dependency confirmed by improved interobserver agreement indicate potential utility of AFI for objective evaluation of left ventricular function during echocardiographic stress test.
